# Explaining Racial Disparities in Amputation Rates for the Treatment of Peripheral Artery Disease (PAD) Using Decomposition Methods

**DOI:** 10.1007/s40615-016-0261-9

**Published:** 2017-02-15

**Authors:** J. A. Mustapha, Bryan T. Fisher, John A. Rizzo, Jie Chen, Brad J. Martinsen, Harry Kotlarz, Michael Ryan, Candace Gunnarsson

**Affiliations:** 1 Metro Health University of Michigan Health, Wyoming, MI USA; 2 Vascular and Endovascular Surgery, The Surgical Clinic, PLLC, Nashville, TN USA; 3 Stony Brook University, Stony Brook, NY USA; 4 University of Maryland, College Park, MD USA; 5 Cardiovascular Systems, Inc., St Paul, MN USA; 6 CTI Clinical Trials and Consulting Services, Inc., 1775 Lexington Avenue, Suite 200, Cincinnati, OH 45212 USA

**Keywords:** Racial disparities, Peripheral artery disease, Amputation, Hospital admission, Multivariate analysis 62Hxx, Estimation 62H12, Hypothesis testing 62H15

## Abstract

**Introduction:**

While studies have documented racial and ethnic disparities in amputation rates for patients with peripheral artery disease (PAD), the importance of specific factors has not been quantified. This research seeks to provide such evidence and to quantify how much of the difference reflects observable versus unexplained factors.

**Methods:**

This study used the nationally representative HCUP inpatient database from 2006 to 2013 for patients with a primary diagnosis of PAD who were either Caucasian, African-American, or Hispanic. Multivariable logistic regression models were estimated to identify the determinants of amputation rates.

**Results:**

Multivariable results revealed that African-Americans and Hispanics are approximately twice as likely to be amputated as are Caucasians. Observed factors in the models collectively account for 51 to 55 % of the disparities for African-Americans and 64 to 69 % for Hispanics. The results suggest that African-Americans and Hispanics have less access to care, because they are being admitted when sicker and more likely on an emergent basis.

**Conclusions:**

Racial and ethnic disparities in amputation rates are substantial, with disease severity and hospital admission source being key factors.

**Electronic supplementary material:**

The online version of this article (doi:10.1007/s40615-016-0261-9) contains supplementary material, which is available to authorized users.

## Introduction

Healthcare disparity is an active area of concern for policymakers. Since the year 2000, the Department of Health and Human Services’ Healthy People initiative has included the elimination of health disparities as an overarching goal. In Healthy People 2020, the stated goal is to ‘*achieve health equity, eliminate disparities, and improve the health of all groups*’ [1]. As reported in the 2014 National Healthcare Quality and Disparities report, significant disparities remain in many areas of healthcare access, quality, and outcomes, including in measures of chronic disease management [2].

Issues relating to racial and ethnic disparity in the treatment, management, and outcomes for patients with peripheral artery disease (PAD) and critical limb ischemia (CLI) have been well documented since the 1990s [3, 4]. Compared to Caucasian patients, several studies have found that African-Americans with PAD are more likely to be amputated and less likely to have their lower limb revascularized either surgically or via an endovascular approach [3–9]. In an early analysis of data from acute-care hospitals in Florida, Huber et al. reported that the incidence of amputation (5.0 vs. 2.5 per 10,000) was higher and revascularization lower (4.0 vs. 7.1 per 10,000) among African-Americans compared to Caucasians, even though the incidence of any procedure for PAD was comparable (9.0 vs. 9.6 per 10,000) [4]. Other studies have reported that the probability of undergoing a revascularization or angioplasty was reduced by 28–49 % among African-Americans relative to Caucasians [3 6]. In a recent study that used multiple logistic regression to control for confounding variables, African-Americans were estimated to be at a 77 % higher risk of lower extremity amputation versus revascularization when compared to Caucasian patients [7]. In a multiple logistic regression analysis of inpatient Medicare data (2003 through 2006), Holman ﻿et al﻿., determined that African-Americans diagnosed with PAD who had undergone a major lower extremity amputation were also significantly less likely than Caucasians to have undergone revascularization (28 % less likely), limb-related admission (19 % less likely), or wound debridement (20 % less likely) in the 2 years prior to amputation in comparison to Caucasian amputees [10]. Newhall et al., using Medicare data (2007–2011), explored geographic variation in amputation-free survival postrevascularization (endovascular or open) for patients diagnosed with PAD and diabetes; amputation-free survival at 2 years ranged from 53.7 % in Savannah Georgia to 76.7 % in Gary Indian for African-Americans and from 64.9 % in Appleton Wisconsin to 83 % in Yakima Washington for Caucasians [11]. Data summarized in a recent (2011) review demonstrates that disparities in pre-amputation care, frequency of amputation in comparison to limb-salvaging procedures, and level (above or below knee) of amputation in minorities as compared to Caucasian patients persist [12]. While revascularization procedures including endovascular interventions have increased over the past two decades, significant racial and ethnic disparities remain in the treatment of patients with PAD and CLI [13].

A recent descriptive study demonstrated the low revascularization and high amputation rates in PAD patients among African-Americans and Hispanics compared to Caucasians on a national level [13]. However, it did not provide multivariate evidence to control for potential confounders, which could bias the estimated associations between race and amputation rates. It is plausible that commonly cited reasons, including racial/ethnic variations in disease or comorbid distribution, differential access to care, racial bias and/or patient preferences (e.g., distrust in the medical system by some in the African-American community), and cultural and/or linguistic barriers, could explain these ongoing disparities [13, 14].

While there is little literature available that explores these underlying issues using multivariate methods, a recent study by Durazzo et al. [7] highlights the complexity of this landscape. In a multivariate logistic regression analysis of NIS data (2002-2008), Durazzo et al. found that the increased risk of undergoing an amputation for an African-American in comparison to a Caucasian rose from 43 to 98 % with the increasing revascularization capacity of the presenting hospital [7]. This seemingly paradoxical result indicates that access to hospitals with greater capacity for limb-salvaging therapies increased racial disparity in treatment. The authors also noted that while the overall odds of being treated with revascularization rather than amputation increased with increasing mean income of the patients’ zip code of residence, the rate of increase was greater for Caucasians than for African-Americans, resulting in increased disparity in relative amputation rates in the wealthier zip codes.

The existing research does not support policy efforts aimed at improving access to limb-saving treatment for African-Americans and Hispanics because the individual importance of these potential factors in explaining the disparities has not been well quantified. Indeed, while the *existence* of racial and ethnic disparities in amputation rates has been well documented, there is a paucity of evidence that attempts to explain the factors behind these disparities. Yet, such evidence is critical from a policy perspective. For instance, it is important to know whether differences in patient health, demographic characteristics, health insurance status, or treatment setting are most responsible for observed differences in revascularization and amputation rates. Without such evidence, any future policy efforts aimed at improving the quantity and quality of care for minority Americans will be hampered.

The present study seeks to bridge several existing gaps in the literature. First, we apply Blinder–Oaxaca decomposition methods to identify and quantify specific factors accounting for racial and ethnic disparities in amputation rates among PAD patients [15, 16]. Second, we quantify the aggregate effects of all observed factors (e.g., patient health, demographic characteristics, health insurance status, and treatment setting) in explaining these disparities. Finally, we estimate how much of the racial and ethnic disparities persist even after accounting for a wide variety of observed factors. This will highlight the limitations in the ability to reduce disparities by affecting observable factors and the potential need to explore other factors that may be more difficult to measure but are nonetheless important in accounting for these differences.

## Methods

This is a retrospective observational study, utilizing the Healthcare Cost and Utilization Project (HCUP) national inpatient database. Descriptive statistics by race were prepared to summarize patient characteristics, comorbidities, and sociodemographic factors for each inpatient visit with a primary diagnosis of PAD. Primary outcomes of interest for this analysis were amputation and revascularization.

### Data Source

Patient visits in the HCUP database from 2006 to 2013, the largest all-payer inpatient care database in the USA, were assessed for eligibility. This database contains data from a family of healthcare databases and related software tools and products developed through a Federal-State-Industry partnership and sponsored by the Agency for Healthcare Research and Quality (AHRQ).

HCUP databases combine the data collection efforts of State organizations, hospital associations, private data organizations, and the Federal government to create a national information resource of patient-level healthcare data. The HCUP is the largest publicly available all-payer inpatient healthcare database in the USA, yielding national estimates of hospital inpatient stays. Unweighted, it contains data from more than 7 million hospital stays each year. Weighted, it estimates more than 36 million hospitalizations nationally [17]. The HCUP database enables research on a broad range of health economics and policy issues, including cost and quality of health services, medical practice patterns, access to healthcare programs, and outcomes of treatments at the national level.

### Study Population

Selection criteria for this study were designed to be as broad as possible while maximizing the likelihood that patients are accurately characterized with respect to having PAD. Inpatient visits meeting the following criteria were eligible for inclusion in the study: (1) patient visits must have a primary diagnosis of PAD (see Online Resource A for complete list of ICD-9 codes); (2) race or ethnic background of interest must be reported; and (3) the patient visit cannot contain a diagnosis for a “traumatic” amputation of a limb: 895.x, 896.x, 897.x.

There are three patient cohorts of interest for this analysis: Caucasians, African-Americans, and Hispanics. Although HCUP categories included Native Americans, Asian or Pacific Islanders, and other, our study restricts the sample to the three main race/ethnic cohorts as stated above. HCUP coding combines race and ethnicity in one data element. If the source supplied race and ethnicity in separate data elements, ethnicity took precedence over race in setting the HCUP value for race. Thus, a patient that was African-American and Hispanic would be counted as Hispanic. Not all state data sources provide information on race and ethnicity. Only hospital visits that had race/ethnicity measures of interest were utilized for this analysis.

### Variable Definitions

Outcome variables used in this analysis included amputation and revascularization procedures. Leg amputations were categorized as follows: any amputation (any part of the leg or foot) or lower leg amputation (below the knee, ankle, foot, or toe). Revascularization was defined as patient visits with a record of the following procedures: peripheral artery bypass graft, peripheral artery angioplasty, peripheral artery stenting, or atherectomy.

Using these criteria for amputation and revascularization, we constructed four outcome variables to investigate the robustness of the results to alternative measures. The first two examine whether a patient received any leg amputation (including lower and upper leg) or whether the patient received a lower leg amputation (e.g., below the knee). Patients receiving no amputation formed the reference cohort in each case. The second set of outcome variables were restricted to patients who either received an amputation or revascularization procedure. In this case, we wished to examine potential disparities among patients who received amputation or revascularization; hence, medically managed patients were excluded from these outcomes. Thus, we constructed a variable measuring whether a patient received an amputation or revascularization procedure at any leg site (e.g., including lower and upper leg) and a corresponding variable indicating whether a patient received amputation or revascularization at a lower limb site. The precise definitions of these variables are provided in the legend to Table 5.

Covariates considered for this analysis include patient demographics (age, gender, health insurance type, and income); hospital visit characteristics (such as admission type, day of week, and number of procedures performed); diagnosis-related group (DRG)-defined disease severity and mortality risk; comorbidities (AHRQ-determined comorbidity measures and calcium risk factors); hospital characteristics (bed size, teaching status, Census Region, urban–rural location); and year of observation. Comorbidity measures were assigned using the AHRQ comorbidity software. The AHRQ comorbidity measures identified coexisting medical conditions that were not directly related to the principal diagnosis or the main reason for admission and were likely to have originated prior to the hospital stay. Comorbidities were identified using ICD-9-CM diagnoses and the DRG in effect on the discharge date.

### Statistical Analyses

Continuous variables were summarized by the mean and standard deviation. Categorical variables were summarized with counts and percentages. Summary data tables were generated by race for the following: patient demographics, hospital visit characteristics, patient comorbidities and risk factors, hospital characteristics, and amputation and revascularization outcomes of interest.

The research relies on the well-known Blinder–Oaxaca decomposition method to ascertain and quantify the factors that contribute to the racial/ethnic disparities in the treatment of PAD patients [15, 16]. This approach, which originated in labor economics [15, 16], has been increasingly applied in recent years to better understand the determinants of racial and ethnic disparities in healthcare utilization, treatment patterns, and outcomes [18]. The decomposition method proceeds by estimating separate equations for amputation rates for each racial cohort of interest. We first compared Caucasians and African-Americans and then Caucasians and Hispanics by estimating logistic regressions predicting amputation rates as

1 $$ \mathrm{PrAM}{\mathrm{P}}^{\mathrm{A}} = {\upbeta_0}^{\mathrm{A}} + {\upbeta_1}^{\mathrm{A}}*{\mathrm{X}}_1 + {\upbeta_2}^{\mathrm{A}}*{\mathrm{X}}_2 $$

2 $$ \mathrm{PrAM}{\mathrm{P}}^{\mathrm{H}} = {\upbeta_0}^{\mathrm{H}} + {\upbeta_1}^{\mathrm{H}}*{\mathrm{X}}_1 + {\upbeta_2}^{\mathrm{H}}*{\mathrm{X}}_2 $$

3 $$ \mathrm{PrAM}{\mathrm{P}}^{\mathrm{C}} = {\upbeta_0}^{\mathrm{C}} + {\upbeta_1}^{\mathrm{C}}*{\mathrm{X}}_1 + {\upbeta_2}^{\mathrm{C}}*{\mathrm{X}}_2 $$

Equation 1 predicts the likelihood of having an amputation among African-Americans, Eq. 2 predicts the likelihood of having an amputation among Hispanics, and Eq. 3 predicts the likelihood of this outcome for Caucasian patients. The *βs* are coefficients to be estimated, and *X*_1_ and *X*_2_ are explanatory variables predicting the likelihood of getting an amputation in these simple models.

Using the results from these models, one may decompose the difference in the mean likelihood of getting an amputation between African-Americans and Caucasians as well as between Hispanics and Caucasians into two components. The first component depicts differences in the *values* of the estimated coefficients (e.g., *βs*). The second component depicts differences in the *levels* of the explanatory variables (e.g., the *Xs*). Using this procedure, one can estimate not only the mean overall difference in the likelihood of amputation by race, but how much of the difference is due to racial differences in the values of the explanatory variables and how much reflects racial differences in how each group responds to those variables (e.g., racial differences in the values of the estimated *βs*). Changes due to values in the *Xs* are interpreted as observed differences while changes in the *βs* are due to unobserved factors. This technique has useful public policy implications because it can inform how much of the differences in amputation rates reflect differences in observable levels of explanatory variables and the relative importance of each one of those variables in affecting the disparity.

## Results

**Fig. 1:**
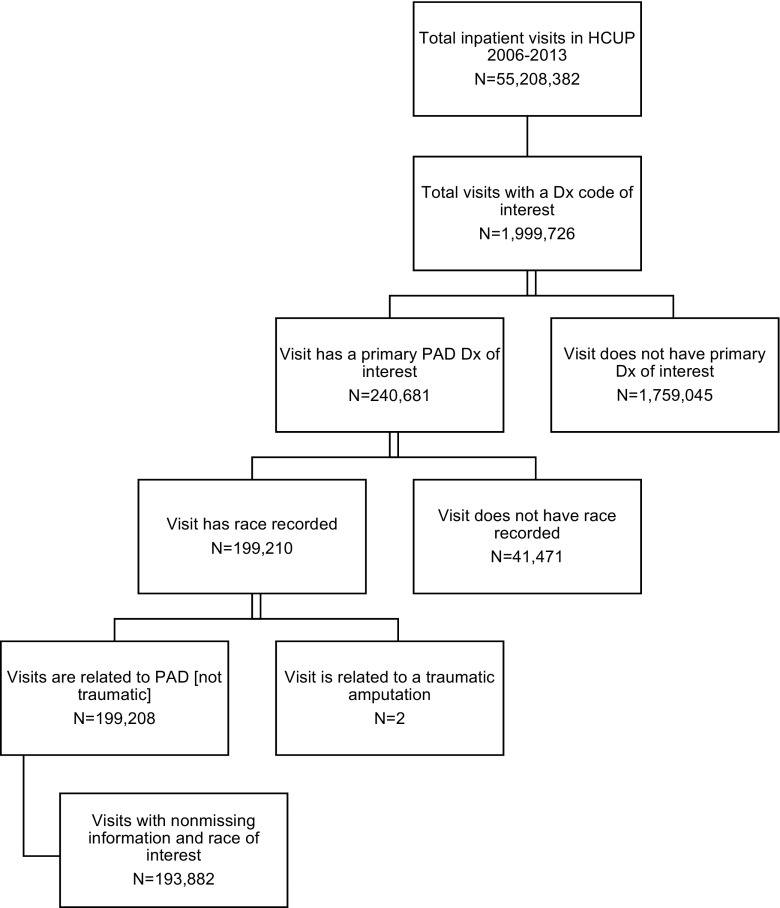
Attrition diagram. *HCUP* Healthcare Cost and Utilization Program; *PAD* peripheral artery disease

For all patient visits meeting the inclusion criteria, there were 143,993 Caucasians, 34,612 African-Americans, and 15,277 Hispanics (see Fig. 1 for full attrition diagram). Patient demographics by race are illustrated in Table 1. Caucasians are generally older, wealthier, and less likely to have Medicaid insurance than are either African-Americans or Hispanics. African-Americans are more likely to be female than are either Caucasians or Hispanics.

**Table 1:** Patient visit demographics by race

	Caucasian		African-American		Hispanic	
	*N*	Percent	*N*	Percent	*N*	Percent
Total visits	143,993		34,612		15,277	
Age (years)^ab^						
Mean	70.3		67.8		69.6	
Std. dev.	11.79		12.20		11.59	
Age category^ab^						
35–44	2020	1.4	762	2.2	253	1.7
45–54	12,874	8.9	4386	12.7	1418	9.3
55–64	30,669	21.3	9127	26.4	3311	21.7
65–74	42,746	29.7	9763	28.2	4722	30.9
75–84	38,113	26.5	7213	20.8	4075	26.7
85+	17,571	12.2	3361	9.7	1498	9.8
Gender^ab^						
Male	84,367	58.6	17,177	49.6	8594	56.3
Female	59,626	41.4	17,435	50.4	6683	43.8
Index year^ab^						
2006	18,869	13.1	4132	11.9	1748	11.4
2007	17,573	12.2	3851	11.1	2174	14.2
2008	19,773	13.7	4384	12.7	2154	14.1
2009	18,433	12.8	3983	11.5	1785	11.7
2010	16,904	11.7	4693	13.6	1880	12.3
2011	18,811	13.1	5160	14.9	2003	13.1
2012	17,154	11.9	4315	12.5	1755	11.5
2013	16,476	11.4	4094	11.8	1778	11.6
Type of health insurance^ab^						
Commercial	28,035	19.5	5169	14.9	2188	14.3
Medicare	103,265	71.7	24,019	69.4	10,471	68.5
Medicaid	7191	5.0	3798	11.0	1872	12.3
Other/unknown	5502	3.8	1626	4.7	746	4.9
Median household income quartile^ab^						
0–25 %	37,461	26.0	18,725	54.1	6986	45.7
25–50 %	39,724	27.6	7277	21.0	3297	21.6
50–75 %	34,458	23.9	4849	14.0	2832	18.5
75–100 %	29,558	20.5	3010	8.7	1688	11.1
Missing	2792	1.9	751	2.2	474	3.1

*Note*: Two-sample *t* tests were used to test continuous variables, and chi-square tests were used to test categorical variables. Due to large sample size and multiple pairwise comparisons, only *p* < .0001 levels are reported

^a^Denote statistically significant difference (*p* < .0001) when the African-Americans are compared to Caucasians

^b^Denote statistically significant difference (*p* < .0001) when Hispanics are compared to Caucasians

Table 2 describes multiple serious issues that could explain the worse outcomes and higher amputation rates for African-Americans and Hispanics. Caucasians are more likely to schedule an elective procedure. African-Americans and Hispanics were more likely to present to the emergency department (ED) for PAD, which is consistent with waiting too long for treatment. Caucasians are more likely to be admitted on weekdays than are either African-Americans or Hispanics. The mean number of chronic conditions is slightly lower for Caucasian than for African-Americans or Hispanics.

**Table 2:** Patient visit characteristics by race

	Caucasian		African-American		Hispanic	
	*N*	Percent	*N*	Percent	*N*	Percent
Total visits	143,993		34,612		15,277	
Admission day of the week^ab^						
Weekday	135,621	94.2	31,999	92.5	14,142	92.6
Weekend	8372	5.8	2613	7.6	1135	7.4
Admission type^ab^						
Emergency	29,559	20.5	10,842	31.3	4757	31.1
Elective	88,750	61.6	17,457	50.4	7516	49.2
Other	22,969	16.0	5771	16.7	2177	14.3
Unknown	2715	1.9	542	1.6	827	5.4
Number of chronic conditions^ab^						
Mean	6.78		7.00		7.00	
Std. dev.	3.01		3.01		2.95	
Number of diagnoses^ab^						
Mean	10.33		10.56		10.50	
Std. dev.	5.24		5.21		5.30	
Number of procedures^ab^						
Mean	3.88		3.74		3.97	
Std. dev.	2.80		2.77		2.92	
Transfer status^a^						
Transferred in	5844	4.1	1572	4.5	637	4.2
Transferred out	17,790	12.4	6369	18.4	1872	12.3

*Note*: Two-sample *t* tests were used to test continuous variables, and chi-square tests were used to test categorical variables. Due to large sample size and multiple pairwise comparisons, only *p* < .0001 levels are reported

^a^Denote statistically significant difference (*p* < .0001) when the African-Americans are compared to Caucasians

^b^Denote statistically significant difference (*p* < .0001) when Hispanics are compared to Caucasians

Table 3 shows a broad summary of the comorbidities that tend to be associated with poor PAD outcomes. Patient comorbidities and risk factors reveal that African-Americans and Hispanics are at substantially higher risk of mortality than are Caucasians. African-Americans and Hispanics have more severe disease as measured by DRG severity and are more likely to have anemia, hypertension, and diabetes than are Caucasians. But Caucasians are more likely to have chronic pulmonary diseases and to be smokers. Regarding geographical differences, African-Americans are more likely to be located in the South with Hispanics more likely to be located in the West than are Caucasians. Racial and ethnic differences by hospital characteristics are relatively minor (Table 4).

**Table 3:** Patient visit comorbid conditions and risk factors

Category	Caucasian		African-American		Hispanic	
	*N*	Percent	*N*	Percent	*N*	Percent
Total visits	143,993		34,612		15,277	
APR DRG mortality^ab^						
Minor	59,964	41.6	11,307	32.7	5138	33.6
Moderate	54,994	38.2	13,847	40.0	6371	41.7
Major	23,246	16.1	7853	22.7	3072	20.1
Extreme	5789	4.0	1605	4.6	696	4.6
APR DRG severity^ab^						
Minor	40,006	27.8	7057	20.4	3286	21.5
Moderate	58,873	40.9	12,794	37.0	6089	39.9
Major	36,419	25.3	11,733	33.9	4800	31.4
Extreme	8695	6.0	3028	8.8	1102	7.2
AHRQ comorbidities						
Alcohol abuse^b^	4100	2.9	947	2.7	256	1.7
Deficiency anemias^ab^	22,929	15.9	9285	26.8	3805	24.9
Obesity^a^	9587	6.7	2571	7.4	1021	6.7
Peripheral vascular disorders^ab^	43,910	30.5	8128	23.5	4360	28.5
Calcium risk factors						
Diabetes^ab^	54,969	38.2	17,255	49.9	9756	63.9
Chronic hypertension^ab^	107,038	74.3	28,704	82.9	12,326	80.7
Chronic renal insufficiency^ab^	26,863	18.7	11,385	32.9	4664	30.5
Smoker^ab^	36,283	25.2	7655	22.1	2143	14.0
Advanced age (≥65)^a^	98,430	68.4	20,337	58.8	10,295	67.4
Aortocoronary bypass status^a^	24,586	17.1	3283	9.5	2539	16.6
History of stroke^ac^	12,723	8.8	4419	12.8	1477	9.7
Dyslipidemia^ab^	67,527	46.9	13,927	40.2	6916	45.3
History of lower limb amputation^ab^	7614	5.3	3592	10.4	1498	9.8

*Note*: Two-sample *t* tests were used to test continuous variables, and chi-square tests were used to test categorical variables. Due to large sample size and multiple pairwise comparisons, only *p* < .0001 levels are reported

*APR DRG* All Patient Refined Diagnosis Related Groups; *AHRQ* Agency for Healthcare Research and Quality

^a^Denote statically significant difference (*p* < .0001) when the African-Americans are compared to Caucasians

^b^Denote statistically significant difference (*p* < .0001) when Hispanics are compared to Caucasians

^c^Denote statistically significant difference (*p* = 0.0006) when Hispanics are compared to Caucasians

**Table 4:** Patient visit hospital characteristics

Category	Caucasian		African-American		Hispanic	
	*N*	Percent	*N*	Percent	*N*	Percent
Total visits	143,993	100	34,612	100	15,277	100
Bed size^ab^						
Small	17,594	12.2	3507	10.1	1553	10.2
Medium	33,908	23.6	8981	26.0	3339	21.9
Large	92,491	64.2	22,124	63.9	10,385	68.0
Location/teaching status^ab^						
Rural	11,260	7.8	2027	5.9	476	3.1
Urban non-teaching	64,894	45.1	12,023	34.7	7340	48.1
Urban teaching	67,839	47.1	20,562	59.4	7461	48.8
Region^ab^						
Northeast	31,254	21.7	6022	17.4	3237	21.2
Midwest	29,273	20.3	5500	15.9	784	5.1
South	59,482	41.3	20,520	59.3	6704	43.9
West	23,984	16.7	2570	7.4	4552	29.8

*Note*: Two-sample *t* tests were used to test continuous variables, and chi-square tests were used to test categorical variables. Due to large sample size and multiple pairwise comparisons, only *p* < .0001 levels are reported

^a^Denote statistically significant difference (*p* < .0001) when the African-Americans are compared to Caucasians

^b^Denote statistically significant difference (*p* < .0001) when Hispanics are compared to Caucasians

Table 5 displays all outcome variables by race. This reveals substantial racial and ethnic disparities. In particular, African-Americans are approximately twice as likely to be amputated as are Caucasians, and Hispanics are about 50 % more likely to be amputated.

**Table 5:** Amputation outcomes by race

	Caucasian		African-American		Hispanic	
	*N*	Percent	*N*	Percent	*N*	Percent
Total visits	143,993	100	34,612	100	15,277	100
Any leg amputation^a^						
Yes	20,802	14.4	9650	27.9	3170	20.8
No	123,191	85.6	24,962	72.1	12,107	79.2
Any leg amputation vs. revascularization^b^						
Any amputation	14,631	10.2	7228	20.9	2283	14.9
Revascularization	93,154	64.7	17,189	49.7	8600	56.3
Excluded	36,208	25.1	10,195	29.5	4394	28.8
Lower leg amputation^c^						
Yes	14,440	10.0	6287	18.2	2403	15.7
No	123,191	85.6	24,962	72.1	12,107	79.2
Excluded	6362	4.4	3363	9.7	767	5.0
Lower leg amputation vs. revascularization^d^						
Lower amputation	9013	6.3	4166	12.0	1612	10.6
Revascularization	93,154	64.7	17,189	49.7	8600	56.3
Excluded	41,826	29.0	13,257	38.3	5065	33.2

^a^Any amputation includes upper leg or lower leg amputation (84.11–84.19)

^b^Any visits with no amputation or revascularization procedure or with both an amputation and revascularization procedure are excluded

^c^Lower leg amputation includes any major or minor lower leg amputation (84.11–84.16). Visits with an upper leg amputation procedure are excluded

^d^Any visits with no lower leg amputation or revascularization procedure or with both a lower leg amputation and revascularization procedure are excluded. Additionally, visits with an upper leg amputation procedure are excluded

Results of multivariable models comparing African-Americans to Caucasians are provided in Table 6. In the interest of brevity, Table 6 only reports those explanatory variables that contributed 5 % or more in terms of accounting for the disparities. Tables showing the effects of all explanatory variables are available from the authors on request. The results in Table 6 indicate that amputation rates remain twice as high for African-Americans compared to Caucasians. Moreover, for African-Americans, observed factors (e.g., all of the variables in the models) collectively account for about 50 to 55 % of the disparities in amputation rates. The most important individual factors accounting for these differences are higher mortality risk and disease severity among African-Americans and a higher chance of ED admission. This suggests that African-Americans have less access to care, because they are being admitted when sicker and more likely on an emergent basis. The corresponding results comparing Hispanics and Caucasians are given in Table 7.

**Table 6:** Decomposition results: Caucasian vs. African-American

	Any leg amputation		Lower leg amputation		Any amputation vs. revascularization		Lower leg amputation vs. revascularization	
Predicted probability (African-American)	0.28		0.20		0.30		0.20	
Predicted probability (Caucasian)	0.14		0.10		0.14		0.09	
Difference	0.13		0.10		0.16		0.11	
Total explained %	50.98		55.13		54.34		55.22	
Individual factor %	%	*p*	%	*p*	%	*p*	%	*p*
Age ≥85							−6.26	0.00
Female	−7.32	0.00	−12.47	0.00	−5.62	0.00	−9.84	0.00
Medicaid							5.19	0.00
Risk of mortality: moderate	−10.25	0.00						
Risk of mortality: major	16.52	0.00			16.47	0.00		
Severity: moderate	−15.33	0.00	−41.92	0.00	−7.25	0.00	−25.73	0.00
Severity: major	35.28	0.00	64.14	0.00	8.59	0.00	24.98	0.00
Severity: extreme	17.84	0.00	30.22	0.00			8.10	0.00
ED admitted	6.34	0.00	6.29	0.00	16.95	0.00	14.46	0.00
Region: south	9.11	0.00	6.22	0.00	8.19	0.00	5.95	0.00
Deficiency anemias	14.17	0.00	17.82	0.00	10.53	0.00	14.44	0.00
Peripheral vascular disorders	5.96	0.00	7.21	0.00	22.45	0.00	27.02	0.00
CRF: diabetes			11.36	0.00			6.81	0.00
CRF: hypertension							5.27	0.00
CRF: dyslipidemia	8.50	0.00	8.56	0.00	10.48	0.00	12.73	0.00
CRF: aortocoronary bypass status	6.69	0.00	6.76	0.00	6.31	0.00	7.01	0.00

*Note*: among the individual factors, we only reported the factors that contributed to 5 % or more to the disparities

*CRF* calcium risk factor, *ED* emergency department

**Table 7:** Decomposition results: Caucasian vs. Hispanic

	Any leg amputation		Lower leg amputation		Any amputation vs. revascularization		Lower leg amputation vs. revascularization	
Predicted probability (Hispanics)	0.21		0.17		0.21		0.16	
Predicted probability (Caucasian)	0.14		0.10		0.14		0.09	
Difference	0.06		0.06		0.07		0.07	
Total explained (%)	69.47		68.06		67.55		63.74	
Individual factor (%)	%	*p*	%	*p*	%	*p*	%	*p*
Female	−5.57	0.00	−8.86	0.00			−9.06	0.00
Medicaid					6.77	0.00	6.90	0.01
Risk of mortality: moderate	−9.79	0.00	0.32	0.53	−5.17	0.00	1.14	0.00
Risk of mortality: major	10.72	0.00			10.85	0.00		
Risk of mortality: extreme			−9.02	0.00				
Severity: moderate	−33.53	0.00	−47.00	0.00	−17.52	0.00	−37.21	0.00
Severity: major	49.04	0.00	58.70	0.00	14.51	0.00	31.16	0.00
Severity: extreme	21.28	0.00	29.77	0.00			5.85	0.00
ED admission	17.12	0.00	14.27	0.00	21.81	0.00	17.65	0.00
Rural hospital	−11.96	0.00	−11.80	0.00	−11.59	0.00	−14.21	0.00
Region: midwest					−10.87	0.04	−13.85	0.05
Region: west					7.19	0.04	13.52	0.01
CRF: anemia	19.82	0.00	17.24	0.00	12.45	0.00	13.34	0.00
CRF: peripheral vasc. dis.	9.18	0.00	8.48	0.00	32.36	0.00	34.57	0.00
CRF: diabetes	29.23	0.00	35.69	0.00	17.61	0.00	30.59	0.00
CRF: renal failure	−9.35	0.00						
CRF: smoking	5.84	0.01			9.86	0.00	9.89	0.00
CRF: dyslipidemia	6.67	0.00	5.34	0.00	9.29	0.00	10.53	0.00

*Note*: among the individual factors, we only reported the factors that contributed to 5 % or more to the disparities

*CRF* calcium risk factor, *ED* emergency department

Hispanics are about 50 % more likely to be amputated than are Caucasians. This result is consistent across amputation measures. For Hispanics, observed factors (e.g., all of the variables in the models) collectively account for about 64 to 69 % of the disparities in amputation rates, which are significantly higher among Hispanics, though not so high as for African-Americans. The most important individual factors accounting for these differences are higher mortality risk and disease severity among Hispanics, a higher chance of ED admission, higher prevalence of diabetes, and anemia. This suggests that, like African-Americans, Hispanics have less access to care, because they are being admitted when sicker and more likely on an emergent basis. Observed factors explain more of the disparities between Caucasians and Hispanics (64 to 69 %) than between African-Americans and Hispanics (51 to 55 %). Still, there is a substantial portion left unexplained.

## Discussion

Persistent racial and ethnic disparities in access to care and medical treatment of PAD/CLI have been well documented in the literature [3–13, 18–20]. Understanding the reasons for such differences and their relative importance is critical for informing policies aimed at reducing or eliminating such disparities. Yet, there is far less evidence on this important avenue of research. This study has sought to help bridge this gap with respect to PAD treatment.

Consistent with prior research, we find substantial disparities in PAD-related treatment patterns between African-Americans and Hispanics compared to Caucasians. Using four measures of leg amputation rates, we find that African-Americans are amputated at twice the rate of Caucasians in every case. Hispanics are amputated at a rate 50 % higher than are Caucasians.

As healthcare providers, we are striving to uncover fixable causes of the lack of fair care to racial and ethnic minority patients. The discrepancies in care led this group to further investigate the root cause of the sustained increased amputation rates among African-Americans and Hispanics. We find evidence suggesting that Caucasians are accessing advanced care in the earlier stages of their disease. In particular, an examination of individual factors associated with these differences finds that being sicker (e.g., higher mortality risk and disease severity) and being admitted through the ED as opposed to direct inpatient admission are the most important individual factors explaining these differences. These factors reflect, in turn, less access to care overall. Patients who have less access will generally present in worse health and are more likely to go to the ED for their care. Thus, we find that less access translates into substantially different treatment patterns, with minorities with PAD receiving much higher amputation rates.

Yet, much of the variation remains unexplained. It is striking that despite specifying quite detailed models, which include a wide variety of patient demographics, comorbidities, hospital characteristics, regional characteristics, and so on, about 50 % of the disparity in amputation rates between African-Americans and Caucasians remains unexplained and approximately 30 % of the variation between Hispanics and Caucasians.

What factors could account for such unexplained differences? While it is possible that some critical patient, demographic, or hospital characteristics have been omitted from our models, this seems an unlikely explanation, given the rich set of factors that we were able to control for. Moreover, it would be quite surprising if adding more of such variables were able to account for a full half of the disparity, as would be required to explain all of the disparities between African-Americans and Caucasians.

A second possibility is that treatment preferences differ by race and ethnicity. For this to be true, however, African-Americans and Hispanics would need to have a greater preference for leg amputation rather than revascularization or medical management compared to their Caucasian counterparts. We are aware of no study that has formally investigated this issue, but this, too, seems unpersuasive. Simply put, most people of any race would likely wish to avoid amputation if medically possible.

A third possibility is that African-Americans and Hispanics are being systematically treated differently—in terms of having higher amputation rates—*even after* controlling for a wide variety of patient, demographic, and hospital characteristics. This phenomenon, known as *statistical discrimination*, has received growing attention in the medical literature [21–23]. It is based upon Bayesian decision theory. According to this theory, physicians decide upon a course of treatment based on their perception of an individual patient’s likely candidacy for that treatment and the past course of treatment for patients sharing similar characteristics such as race. If—perhaps because of poorer communication with their minority patients—physicians have less information about their individual health states and likelihood of responding to a specific treatment, they will place greater weight on the treatment patterns received in the past by that group. And if those past treatment patterns called for relatively high amputation rates—as in the case of minorities—the pattern gets repeated. Of note in this regard, Newhall et al. [11] find that, among PAD patients who had been revascularized, African-Americans were significantly more likely to require subsequent amputation than their Caucasian counterparts were. Perhaps a prior belief that long-term outcomes are on average worse for revascularized African-Americans leads to more aggressive amputation rates for this group. In any event, statistical discrimination provides a plausible explanation for the large unexplained disparities in amputation rates between Caucasians and minority patients that warrants further study.

Moreover, statistical discrimination helps explain seemingly paradoxical results regarding racial and ethnic disparities in amputation rates that have been reported in the literature. Thus, Durazzo et al. [7] report that racial and ethnic disparities increase among hospitals where revascularization capabilities are greatest. The paradox here is that greater availability of revascularization exacerbates the disparities. Seen through the lens of statistical discrimination, however, this finding becomes less surprising. First, among hospitals having little or no ability to revascularize, amputation rates should be similar since revascularization is not a viable alternative. In this case, statistical discrimination—systematically revascularizing Caucasians more than minorities—is not possible. But when revascularization is readily available, physicians can statistically discriminate, and Durazzo et al. find that Caucasians are substantially less likely to get amputated.

Research has demonstrated that patient–physician communication with African-Americans differs significantly from their Caucasian counterparts:

*Physicians were 23 % more verbally dominant and engaged in 33 % less patient-centered communication with African American patients than with White patients. Furthermore, both African American patients and their physicians exhibited lower levels of positive affect than White patients and their physicians did* [24].

Moreover, poor communication between physicians and their minority patients has been cited as a potentially important factor contributing to racial and ethnic disparities in other contexts [25, 26]. Kwolek et al. [27] argue that a more diverse vascular surgery workforce is needed to help address racial and ethnic disparities in treatment patterns. Greater diversity should help to improve physician–patient communication. As statistical discrimination is itself likely rooted in a lack of communication between physicians and their minority patients, the potential for improved communication to reduce disparities in amputation rates would seem to be quite large. While these are intriguing possibilities, the actual extent to which statistical discrimination affects differences in PAD treatment patterns requires further research.

### Study Limitations

This study has some limitations that must be acknowledged. One limitation common to all retrospective research is the lack of an experimental design. But HCUP data have been widely used to evaluate the association between treatments and clinical outcomes, which is particularly valuable when a portrayal of patient experience outside the controlled setting of the clinical trial is desired. Because HCUP includes a rich variety of variables, including patient demographic characteristics, comorbid conditions, and hospital characteristics, we believe that important confounding factors may be controlled for, resulting in reliable estimates of the impacts of race and ethnicity on the outcomes of interest. Moreover, both discharge and hospital weights are available in HCUP, enabling one to generate nationally representative effects of race and ethnicity on these outcomes. But while our analysis has identified factors associated with racial and ethnic disparities in amputation rates, our lack of an experimental design precludes making causal inferences. Another limitation is that our inclusion criteria to identify PAD candidates for amputation or revascularization were based on a primary diagnosis code for PAD. It is possible that some of these patients were not candidates for any invasive treatment despite having this primary diagnosis. Finally, while statistical discrimination provides a plausible explanation for the unexplained racial and ethnic differences in amputation rates, this must be viewed with caution as it is possible that other factors may be at work as well.

## Conclusion

Racial and ethnic disparities in amputation rates are substantial, with disease severity and hospital admission source being key factors. As the population ages and comorbidities rise, these disparities may accelerate unless access among minorities improves. Moreover, because some 30 to 50 % of these disparities remain unexplained by access or other observed factors, substantial disparities could persist despite improvements in access. Identifying the precise factors causing these large unexplained variations is an important direction for further study.

## Electronic supplementary material

Below is the link to the electronic supplementary material.

ESM 1 (DOCX 23 kb)
