# Supplementary material for: Explaining Racial Disparities in Amputation Rates for the Treatment of Peripheral Artery Disease (PAD) Using Decomposition Methods
Source: J Racial Ethn Health Disparities. 2017 Feb 15;4(5):784–95. doi: 10.1007/s40615-016-0261-9 (PMC5626799; doi:10.1007/s40615-016-0261-9)
Supplement: Supplementary file 1 — (DOCX 23 kb) [file 40615_2016_261_MOESM1_ESM.docx]

**Title: Explaining Racial Disparities in Amputation Rates for the Treatment of Peripheral Artery Disease (PAD) using Decomposition Methods**

**Journal of Racial and Ethnic Health Disparities**

Authors: Jihad Mustapha, MD; Bryan T. Fisher Sr., MD; John A. Rizzo, Ph.D.; Jie Chen, Ph.D.; Brad J. Martinsen, Ph.D.; Harry Kotlarz, MBA; Michael Ryan, MS; Candace Gunnarsson Ed.D.

**Corresponding Author**

Candace Gunnarsson EdD

CTI Clinical Trial and Consulting Services, Inc.

cgunnarsson@ctifacts.com

**Online Resource A:** International Classification of Diseases, 9th revision, Clinical Modification (ICD-9-CM) for Primary Diagnosis of Peripheral Artery Disease (PAD)

| **Peripheral Artery Disease Native Vessels** | |
| --- | --- |
| 440.20 | Atherosclerosis of native vessels of the extremities, unspecified |
| 440.21 | Atherosclerosis of native vessels of the extremities, with intermittent claudication |
| 440.22 | Atherosclerosis of native vessels of the extremities, with rest pain |
| 440.23 | Atherosclerosis of native vessels of the extremities with ulceration |
| 440.24 | Atherosclerosis of native vessels of the extremities with gangrene |
| 440.29 | Atherosclerosis of native vessels of the extremities, other |
| **Peripheral Artery Disease with Previous Bypass Graft** | |
| 440.30 | Atherosclerosis of bypass graft of the extremities, unspecified graft |
| 440.31 | Atherosclerosis of bypass graft of the extremities, autologous vein bypass graft |
| 440.32 | Atherosclerosis of bypass graft of the extremities, nonautologous biological bypass graft |
| **Other Codes of Interest with Respect to Peripheral Artery Disease** | |
| 443.9 | Peripheral vascular disease, unspecified |
